# Evaluation of a semiautomated App Store analysis for the identification of health apps for cardiac arrhythmias

**DOI:** 10.1007/s00399-023-00947-2

**Published:** 2023-06-28

**Authors:** Dennis Lawin, Ute von Jan, Evgenii Pustozerov, Thorsten Lawrenz, Christoph Stellbrink, Urs-Vito Albrecht

**Affiliations:** 1 Department of Digital Medicine, Medical Faculty OWL, Bielefeld University, Bielefeld, Germany; 2 Department of Cardiology and Intensive Care Medicine, University hospital OWL of Bielefeld University, Campus Klinikum Bielefeld, Bielefeld, Germany; 3 Peter L. Reichertz Institute for Medical Informatics of TU Braunschweig and Hannover Medical School, Hannover Medical School, Hannover, Germany

**Keywords:** Digital Medicine, Arrhythmias, Cardiology, Health apps, Mobile health, Digitale Medizin, Arrhythmien, Kardiologie, Gesundheits-Apps, Mobile Gesundheit

## Abstract

**Background:**

Smartphone apps are increasingly utilised by patients and physicians for medical purposes. Thus, numerous applications are provided on the App Store platforms.

**Objectives:**

The aim of the study was to establish a novel, expanded approach of a semiautomated retrospective App Store analysis (SARASA) to identify and characterise health apps in the context of cardiac arrhythmias.

**Materials and methods:**

An automated total read-out of the “Medical” category of Apple’s German App Store was performed in December 2022 by analysing the developer-provided descriptions and other metadata using a semiautomated multilevel approach. Search terms were defined, based on which the textual information of the total extraction results was automatically filtered.

**Results:**

A total of 435 of 31,564 apps were identified in the context of cardiac arrhythmias. Of those, 81.4% were found to deal with education, decision support, or disease management, and 26.2% (additionally) provided the opportunity to derive information on heart rhythm. The apps were intended for healthcare professionals in 55.9%, students in 17.5%, and/or patients in 15.9%. In 31.5%, the target population was not specified in the description texts. In all, 108 apps (24.8%) provided a telemedicine treatment approach; 83.7% of the description texts did not reveal any information on medical product status; 8.3% of the apps indicated that they have and 8.0% that they do not have medical product status.

**Conclusion:**

Through the supplemented SARASA method, health apps in the context of cardiac arrhythmias could be identified and assigned to the target categories. Clinicians and patients have a wide choice of apps, although the app description texts do not provide sufficient information about the intended use and quality.

## Abstract (de)

**Hintergrund:**

Smartphone-Apps werden von Patient*innen und Ärzt*innen zunehmend für medizinische Zwecke genutzt. Zahlreiche Anwendungen sind in den App-Stores verfügbar.

**Ziel der Arbeit:**

Ziel dieser Studie war es, über einen neuartigen und erweiterten Ansatz einer halbautomatischen retrospektiven App-Store-Analyse (SARASA) Gesundheits-Apps im Zusammenhang mit kardialen Arrhythmien zu identifizieren und zu charakterisieren.

**Material und Methoden:**

Im Dezember 2022 wurde eine automatisierte Auslese der gesamten Kategorie „Medizin“ des deutschen Apple-App-Stores durchgeführt. Dafür wurden die von den Entwicklern stammenden Beschreibungstexte und andere Metadaten in einem halbautomatischen mehrstufigen Ansatz analysiert. Die textlichen Informationen aller Extraktionsergebnisse wurden anhand definierter Suchbegriffe automatisch gefiltert.

**Ergebnisse:**

Unter 31.564 Apps wurden 435 mit einem Anwendungszweck im Kontext kardialer Arrhythmien gefunden. Von diesen dienten 81,4 % der Bildung, der Entscheidungshilfe oder dem Krankheitsmanagement und 26,2 % boten (zusätzlich) die Möglichkeit, Informationen über den Herzrhythmus abzuleiten. Die Anwendungen richteten sich in 55,9 % der Fälle an medizinisches Fachpersonal, in 17,5 % an Studierende und/oder in 15,9 % an Patient*innen. In 31,5 % der Beschreibungstexte war die Zielgruppe nicht angegeben. Insgesamt 108 Apps (24,8 %) enthielten einen telemedizinischen Behandlungsansatz. Während 83,7 % der Beschreibungstexte keinerlei Angaben zum Medizinproduktstatus enthielten, wurde in 8,3 % ein Medizinproduktstatus bejaht und in 8,0 % ein solcher verneint.

**Schlussfolgerung:**

Durch die erweiterte SARASA-Methode konnten Apps im Kontext kardialer Arrhythmien identifiziert und charakterisiert werden. Heutzutage steht eine große Auswahl an Apps für Kliniker*innen und Patient*innen zur Verfügung. Leider liefern die Beschreibungstexte häufig keine ausreichenden Informationen über Verwendungszweck und Qualität.

## Introduction and background

Due to the technological advances of recent years and the extensive use of smartphones and smartwatches, efforts have grown to utilise apps for medical purposes [18]. Health apps are poised to take on significant importance as a vehicle for health guidance and remote data acquisition of digital biomarkers [7]. Thus, several manufacturers provide numerous applications with a health purpose on the App store platforms [1].

While some applications address healthcare professionals and serve as educational or disease-management tools, others are intended for patients [1]. Targeting patients, the opportunity to remotely derive information on the patient’s heart rhythm, which enables the embedment into telemedicine treatment approaches, raised interest in the utilisation of health apps in the field of cardiac arrhythmias [9]. However, concerns about quality and safety exist and comprise loss of data privacy, poor data management, misdiagnosis by unvalidated sensors, and lack of evidence for improving medical endpoints [6]. Beyond this, due to the considerable increase in available applications in recent years, it remains difficult to find applications that meet one’s needs and fulfill quality claims [2].

Thus, methods are required to identify commercially available health apps and obtain information on purpose, target group, costs, certification as a medical product, and other quality distinctions [2]. Recently, we developed and published a semiautomated retrospective App Store analysis (SARASA) to identify and characterise health apps available on Apple’s (Cupertino, CA, USA) App Store and applied it to data from the German storefront [2, 3]. However, there have been some changes to the extent of the apps listed on the App Store’s overview pages since the inception of the methodology, necessitating an expanded approach of SARASA for obtaining a more comprehensive list of apps than would have been possible with our initial read-out methodology. Here, we present the first application of this novel approach for identifying health apps in the context of cardiac arrhythmias from Apple’s German App Store and their assignment to predefined categories.

## Study design and investigation methods

### App store read-out.

The initial description of SARASA has been published previously [2, 3] but was expanded for this work using a novel approach to improve the hit rate. In summary, the algorithm analyses the developer-provided descriptions and other metadata in the German App Store using a semiautomated multilevel approach [2, 3]. The first step is an automated total read-out of the “Medical” App Store category. For this analysis, the read-out was performed between November 30th and December 3rd, 2022. The search was limited to apps assigned to the “Medical” category (primary or secondary category).

The read-out was conducted by accessing the alphabetical listings of the applications provided by Apple on the country-specific websites of the App Store by using a script-based approach via the “iTunes Search APIs” [21]. Surprisingly, some known apps were not included in the initial read-out. Thus, we modified our method to obtain a more comprehensive dataset by separately parsing the alphabetical pages for apps starting with lower case letters and special characters, followed by an additional evaluation of the manufacturer’s store pages (stratified by device categories such as iPad or iPhone) of those manufacturers with at least one listed app in the initial read-out. For the results obtained from the manufacturers’ pages, we also had to filter out non-health apps, as these pages also listed entries for other store categories. Using the language detector of the cld2-bibliography [13], we excluded apps that did not have German or English as the main language in their description texts.

### Identification of apps in the context of cardiac arrhythmias.

To identify only those apps related to cardiac arrhythmias, we subsequently defined search terms based on which the textual information of the total extraction results was automatically filtered by SARASA. We used Perl notations based on the regular expressions as used in “R” for the description of the search terms to allow for capture of different combinations of words [22]. The filtering process was conducted by an automated analysis considering the developer-provided descriptions as well as other metadata of the applications (information on the manufacturer, description texts, costs, requirements for the operating systems, evaluations by users, and the date of publication or actualisations). The used search terms are summarised in Table 1. Initially, the terms “(?<!(om|im|ro|l ))puls(?!(e of smart|ed|at|tar|ier|e practice|nitz|e studio|e.com))”, “heart[ ]*beat”, “herzschlag”, “herz[ -]*frequ”, “heart[ -]*rate” were included but then waived due to a high number of mismatches. Additionally, terms related to veterinary medicine were defined as exclusion criteria (Table 1). The resulting applications, including their metadata, were available for further investigation and categorisation.

**Table 1:** Search terms used for the identification of apps in the context of cardiac arrhythmias and absolute number of hits in the “Medical” category

Search term	Absolute number of hits in the “Medical” category
Heart[ -]*r[h]*yt[h]*m	24
Herz[ -]*r[h]*yt[h]*m	16
Atrial fibrillation	63
Vorhof[-]*flimmern	16
Ele[ck]+tro[ck]+ardiogra	74
(?<!(se|sp))e[ck]+g	318
Tachy(?!(pn|ben|os))	48
Brady(arrhyt|arhyt|card|kard)	28
Photoplethysmograph	3
(?<!(i|u|a))ppg	10
Ar[rh]*yt[h]*m	78
Ele[ck]+trophysiol	24
\bep\b	11
Afib	15
Vhf	2
Cha[2]*ds[2]*[ -]vasc	15
Anti[ -]*[ck]+oagulat	30
Total number of unique apps	479

Multiple counting allowed in case of more than one fitting search term per app

“?<!(om|im|ro|l ))puls(?!(e of smart|ed|at|tar|ier|e practice|nitz|e studio|e.com))”, “heart[ ]*beat”, “herzschlag”, “herz[ -]*frequ”, “heart[ -]*rate” were waived to avoid mismatches

Exclusion terms: “\bcat[s]*\b”, “\bkatze[n]*\b”, “\bdog[s]*\b”, “\bhund[esn]*\b”, “\bhorse[s]*\b”, “\bpferd[esn]*\b”, “\bequine\b”, “\bfeline\b”, “\bcanine\b”

### Manual categorisation.

Subsequently, we manually reviewed the generated results to determine whether they really met the criteria for inclusion. Apps that were not found to fulfill the requirements for inclusion were removed. The remaining apps were categorised regarding purpose (“education/decision support/disease management” and/or “derivation of electrocardiogram [ECG]/photoplethysmography [PPG]/other information on the heart rhythm”), target group (“healthcare professionals” and/or “students” and/or “patients” or “target population not specified”), telemedicine approach (“yes” or “no”) and information on medical product status disclosed in the description text (“app is a medical product which is indicated by the description text” or “app is NOT a medical product which is indicated by the description text” or “medical product status not indicated by the description text”). These categories, including detailed definitions, are summarised in Table 2.

**Table 2:** Categories and their detailed definitions used for further structuring of the health apps

Category		Definition
**Purpose**	Education/decision support/disease management	Apps that are involved in the process of teaching or learning for better understanding of the healthy and the sick, intended as tools supporting clinical decisions, or serving as tools for better management of disease treatment/prevention. Due to the smooth transition of those sub-categories, they were not separated
	*And/or*	
	Derivation of ECG/PPG/other information on the heart rhythm	Apps that provide the technical possibility to derive current information on the user’s heart rhythm. This could be enabled by either ECG, or detecting the pulse wave by PPG, or other technical solutions for derivation of the heart rhythm
**Target group**	Healthcare professionals	People who have received special training for services that involve caring for people’s health (according to [20])
	*And/or*	
	Students	People who are learning at a training facility, college or university (according to [20])
	*And/or*	
	Patients	People who are receiving medical care or who are cared for by a particular doctor or dentist or other healthcare professional when necessary (according to [20])
	*Or*	
	Target population not specified	The description text does not include any information on the target population
**Telemedicine**	Yes	The management of disease, by sending information from one place to another by computer, video, etc. (according to [20])
	*Or*	
	No	The description text does not provide any evidence of telemedicine treatment approaches
**Medical product status**	App is a medical product which is indicated by the description text	The app is indicated as a medical product approved by official institutions. This information is provided by the description text
	*Or*	
	App is NOT a medical product which is indicated by the description text	The description text particularly reveals that the app is not intended for medical purposes
	*Or*	
	Medical product status not indicated by the description text	There is not any information regarding the medical product status in the description text

*ECG* electrocardiogram, *PPG* photoplethysmography

### Analysis.

The apps related to cardiac arrhythmias were assessed with respect to purpose, target group, telemedicine approach, and information on medical product status. Furthermore, we investigated information on costs, length of the description texts, counts of user ratings, and average user ratings. By analysing the average user ratings, we identified the top-rated health apps in the specific category. Therefore, those apps having at least one evaluation were firstly assorted to the absolute rating counts and only the apps of the upper quartile or upper median (latter in case of < 10 apps related to the upper quartile) considered for ranking.

## Results

The initial read-out of the category “Medical”, obtained between November 30th (10:21:25 p.m.) and December 3rd 2022 (9:00:08 p.m.), found 28,970 apps. By parsing the manufacturer pages, we identified another 2594 apps which resulted in overall 31,564 applications in the “Medical” category at that time. Of those, for 22,713 apps (72.0%), the primary category was “Medical”, while for 8849 apps (28.0%), “Medical” was only assigned as a secondary category. By considering only those apps with German or English description texts, 22,674 apps remained for further analysis. After applying the German and English language search terms, exclusion of duplicates, as well as utilisation of the above-mentioned exclusion terms, 479 apps (2.1%) remained with a supposed context in cardiac arrhythmias. The number of hits per search term is shown in Table 1. After the manual review process, a further 44 apps were excluded since they were mismatches and unrelated to cardiac arrhythmias.

**Fig. 1:**
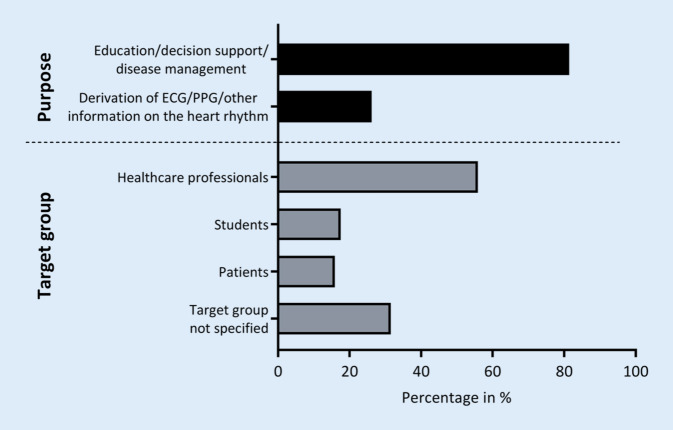
Purpose and target group of the 435 health apps in the context of cardiac arrhythmias. *ECG* electrocardiogram, *PPG* photoplethysmography

**Fig. 2:**
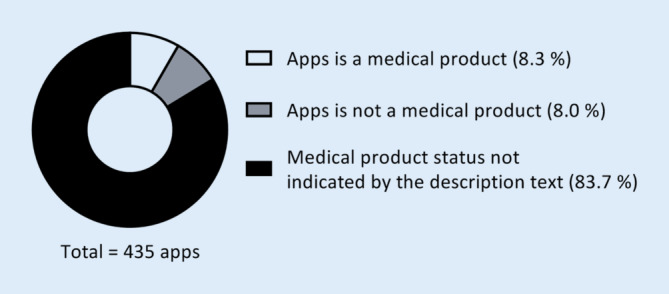
Medical product status based on the information disclosed in the description texts

The remaining 435 apps were manually categorised according to the above-described scheme (Table 2). In all, 354 apps (81.4%) were found to deal with education, decision support, or disease management, and 114 apps (26.2%) (additionally) provided the opportunity to derive information on the heart rhythm (Fig. 1). Most of the apps were identified to be intended for healthcare professionals (243 apps; 55.9%; Fig. 1). Students and patients were addressed in 76 (17.5%) and 69 (15.9%) apps, as indicated by the description texts, respectively. In 137 apps (31.5%), the target population was not specified in the description texts. We found 108 apps (24.8%) providing a telemedicine treatment approach. Most of the description texts did not reveal any information on medical product status (364 apps; 83.7%). Based on the information provided in the description texts, we found 36 apps (8.3%), that stated to have medical device status, and 35 apps (8.0%) explicitly denying this status (Fig. 2). The median length of the description texts was 1410 characters, including spaces and characters related to formatting (min.: 65; max.: 4021). Of the 435 apps related to cardiac arrhythmias, 311 (71.5%) were free of charge. The median cost for the other apps was 5.99 € (Min.: 1.19; Max.: 1199.99).

**Fig. 3:**
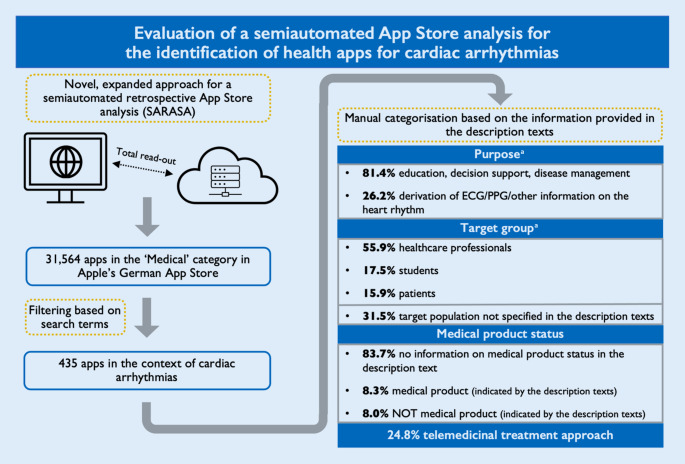
Graphical illustration of the methods and results. *ECG* electrocardiogram, *PPG* photoplethysmography. ^a^ Multiple counting allowed

A total of 143 apps (32.9%) had customer ratings. These had a median count of five ratings (Min.: 1 rating; Max.: 89,069 ratings). The median rating score of apps with at least one rating was 4.43 (out of 5 achievable). The top-rated health apps for the specific categories are shown in Table 3. Of note, those apps having at least one evaluation were firstly sorted to the absolute rating counts, and only the apps of the upper quartile or upper median (latter in case of < 10 apps related to the upper quartile) were considered for the ranking. Our methods and findings are summarised in a graphical illustration in Fig. 3.

**Table 3:** Top-10 ranking of customer ratings of health apps in the specific category

	Name	Release date of latest version	Price in €	Average user rating	Average rating count	Is MP status disclosed in the description text?	Telemetry
**Education/decision support/disease management for physicians**							
*1*	epTools	2022-10-19	23.99	4.87	46	No	No
*2*	MDCalc Medical Calculator	2022-09-12	0.00	4.75	815	No	No
*3*	RetterTool	2022-08-09	0.00	4.74	31	No	No
*4*	DGK Pocket-Leitlinien	2022-03-28	0.00	4.70	706	Yes, MP	No
*5*	CoaguSafe—Antikoagulantien	2021-05-16	3.49	4.65	37	Yes, no MP	No
*6*	INSIGHT HEART	2022-11-24	3.49	4.64	338	No	No
*7*	ESC Pocket Guidelines	2022-10-04	0.00	4.61	259	Yes, MP	No
*8*	Rettungsdienst App	2020-03-19	9.99	4.61	623	No	No
*9*	Calculate by QxMD	2022-08-23	0.00	4.60	197	No	No
*10*	DAPT Advisor	2022-01-10	0.00	4.57	95	No	No
**Education/decision support/disease management for students**							
*1*	Cardio Ex	2022-11-03	0.00	5.00	9	No	No
*2*	Eko academy	2021-07-03	0.00	4.77	13	Yes, MP	No
*3*	INSIGHT HEART	2022-11-24	3.49	4.64	338	No	No
*4*	MediCode-ACLS, PALS, BLS, CPR	2022-11-30	0.00	4.54	13	No	No
*5*	INSIGHT HEART Lite	2022-11-24	0.00	4.44	16	No	No
*6*	EKG für Ärzte	2022-11-06	0.00	4.35	298	No	No
*7*	ECG EKG Interpretation Mastery	2022-05-05	0.00	4.17	6	No	No
*8*	EKG Pro für Ärzte	2022-11-06	23.99	4.14	7	No	No
*9*	Anatomist—Anatomy quiz Game	2019-05-18	0.00	3.83	12	No	No
*10*	Heart Health: Heart Healthy Living Facts & Tips	2017-07-21	0.00	3.78	9	Yes, no MP	No
**Education/decision support/disease management for patients**							
*1*	INSIGHT HEART	2022-11-24	3.49	4.64	338	No	No
*2*	Withings Health Mate	2022-10-25	0.00	4.45	89,069	No	Yes
*3*	INSIGHT HEART Lite	2022-11-24	0.00	4.44	16	No	No
*4*	Curalie	2022-11-16	0.00	4.40	40	Yes, MP	Yes
*5*	Blutdruck—SmartBP	2022-11-18	0.00	4.31	1733	No	Yes
*6*	Qardio Herzgesundheits-App	2022-11-24	0.00	4.28	700	Yes, MP	Yes
*7*	Cardiogramm: Heart Rate Monitor	2022-10-28	0.00	4.12	338	No	No
*8*	iATROS—App für Herzpatienten	2022-11-26	0.00	3.96	24	Yes, MP	Yes
*9*	Heart Health: Heart Healthy Living Facts & Tips	2017-07-21	0.00	3.78	9	Yes, no MP	No
*10*	Elektronische Gesundheitskarte	2021-06-02	0.00	3.62	26	No	No
**Including the derivation of ECG/PPG/other information on the heart rhythm for patients**							
*1*	MedKitDoc	2022-11-02	0.00	5.00	15	Yes, MP	Yes
*2*	Kardiograph Klassik	2019-10-14	1.19	4.60	1788	No	No
*3*	Withings Health Mate	2022-10-25	0.00	4.45	89,069	No	Yes
*4*	Kardiograph	2019-10-14	2.49	4.43	502	Yes, no MP	No
*5*	Blutdruck—SmartBP	2022-11-18	0.00	4.31	1733	No	Yes
*6*	Qardio Herzgesundheits-App	2022-11-24	0.00	4.28	700	Yes, MP	Yes
*7*	Kardia	2022-11-30	0.00	4.26	175	Yes, MP	Yes
*8*	Cardiogram: Heart Rate Monitor	2022-10-28	0.00	4.12	338	No	No
*9*	Preventicus Heartbeats	2022-11-02	0.00	4.01	545	Yes, MP	Yes
*10*	iATROS—App für Herzpatienten	2022-11-26	0.00	3.96	24	Yes, MP	Yes

Those apps having at least one evaluation were firstly assorted to the absolute rating counts and only the apps of the upper quartile or upper median (latter in case of < 10 apps related to the upper quartile) considered for ranking

*E* English, *ECG* Electrocardiogram, *G* German, *Lang* Language, *MP* medical product, *PPG* Photoplethysmography

## Discussion

The technical advances of the past decade led to tremendous growth in the mobile applications market. In the past, there were efforts to gather the full extent of apps provided for the common mobile platforms and develop tools for further characterisation concerning particular features [2, 4, 11, 19]. However, those efforts were often limited to incomplete read-outs and imprecise classifications [2, 4, 11, 19]. Via the novel, expanded approach for our semiautomated method SARASA, we were able to identify overall 31,564 apps in the “Medical” category in Apple’s German App Store. We established an automated identification of apps related to cardiac arrhythmias based on search terms and found 479 apps. Of those, only 9.2% were found not to fit the purpose, necessitating manual exclusion. This indicated a good accuracy of the search algorithm and a proper selection of the search terms. As a result, a surprisingly high number of 435 apps remained in the specific context of cardiac arrhythmias.

Whether healthcare professionals or patients, users commonly rely on the information from the description texts when choosing a suitable app. However, we found that 31.5% of the description texts did not even specify the target population. This may result in misuse of these applications on the one hand or in users refraining from downloading an app on the other hand.

Besides, many of the applications identified aimed to be instantly integrated into patient care processes, thus meeting the definition of a medical product. For the European Union, such applications have to provide evidence that the requirements of the Regulation (EU) 2017/745 of the European Parliament have been fulfilled [14]. After completing the conformity assessment, the manufacturers can attach the CE certificate to their applications [14]. In the United States, applications are approved as medical products by the U. S. Food and Drug Administration (FDA) [8]. The designation, whether a health app has been approved as a medical product or not, is highly relevant for the users and should be disclosed on the download platforms. However, according to other publications, the proportion of apps providing information on the medical product status in the description text was negligible [3]. In our analysis, only in 16.3% of the description texts did the manufacturers provide information about whether the app had medical product status (or not).

Albeit some may consider the CE certificate and FDA-clearance as quality features for medical products, these labels are rather indicators of compliance with conformity requirements that allow for market participation of medical products. They are not to be seen as quality assessments [3]. There have been some efforts to further evaluate health apps regarding quality issues. Previously, we and others worked out quality criteria for software in the context of health apps [1, 5, 15, 16]. Nevertheless, the manufacturers of medical apps are still not asked to follow a standardised quality assessment. In our opinion, an EU-wide standard is required to allow for objective and reliable quality evaluation of medical apps [1]. Due to the lack of such evaluations, users often rely on the average customer ratings to choose an application that fits their purposes. This must be seen in a critical light, but ratings are one of the few information points that can be used in coming to a decision. To provide some clinical implications, we identified the top-rated apps for the most common purposes in the context of cardiac arrhythmias (Table 3).

Nowadays, the technical advances in the field of ECG and PPG allow for remote monitoring of a patient’s heart rhythm by using smartphones or smartwatches alone or in combination with coupled sensors [9, 17]. Such applications may be helpful in diagnosing rhythm disorders in symptomatic subjects, for screening, or to follow-up patients after receiving antiarrhythmic therapy [10, 17]. Health apps for diagnosing cardiac arrhythmias are increasingly accepted [12]. In a recently conducted survey, physicians predominantly saw the advantages of using wearable rhythm devices in daily practice [12]. Although the cardiological societies have published clinical advisories for health apps in the context of cardiac arrhythmias, they avoid explicitly recommending certain manufacturers and products [10, 17]. Due to the high number of 114 apps identified in our analysis to (additionally) provide the opportunity to derive information on the heart rhythm, it is challenging for physicians to maintain an overview over which applications are approved as medical products and fulfill the required quality criteria for the particular purpose.

## Limitations

Our study has some imitations. Firstly, the read-out was limited to Apple’s App Store, and thus, applications only offered on other platforms are missing. Secondly, even with our expanded read-out methodology, some apps were known to be available in the store but were still missing in our acquired dataset (e.g., Fibricheck, Qompium Inc. Hasselt, Belgium). This may be due to several factors. On the one hand, Apple seems to include only apps conforming to specific (unknown) criteria on the store overview pages, probably related to an app’s performance on the store. Similarly, even via the manufacturers’ store pages (stratified by device category), there may be apps for the “Medical” category that we were unable to find. These manufacturer pages only list up to 100 apps per device category, even for manufacturers with considerably more apps in the initial read-out. Besides, there may be some manufacturers with no apps at all in the initial read-out, and we may thus have missed apps for those manufacturers as well. The high market dynamics and the associated fluctuation of apps on offer during the read-out process may also be aggravating factors. Due to the long duration for the complete read-out (aside from network speeds, also attributable to limitations in the number of requests allowed per minute by Apple’s servers), there were a few apps specified in the original lists (obtained from the overview pages), but that were missing for the metadata read-out later on. For our read-out, this was true for two apps. Thirdly, some health apps in the context of cardiac arrhythmias may have been missed due to not matching any of the keywords chosen to identify eligible apps.

## Practical conclusion

- Utilising the supplemented SARASA method, health apps in the context of cardiac arrhythmias could be identified and assigned to the target categories.
- Clinicians and patients have a wide choice of apps, although the app description texts do not provide sufficient information about the intended use and quality.

## Declarations

For this article no studies with human participants or animals were performed by any of the authors. All studies mentioned were in accordance with the ethical standards indicated in each case.
